# A survey on doctors’ cognition of depression in patients with epilepsy

**DOI:** 10.1002/brb3.2232

**Published:** 2021-06-04

**Authors:** Weifeng Peng, Jing Ding, Shaokang Zhan, Xin Wang, the epilepsy group affiliated to Neurology Committee, Shanghai Medical Association

**Affiliations:** ^1^ Department of Neurology Zhongshan Hospital Fudan University Shanghai China; ^2^ Institute of Statistics and Public Health Shanghai Medical College Fudan University Shanghai China; ^3^ Department of The State Key Laboratory of Medical Neurobiology and MOE Frontiers Center for Brain Science Institutes of Brain Science Fudan University Shanghai China

**Keywords:** comorbidity, depression, doctor survey, epilepsy

## Abstract

**Objective:**

This survey aimed to assess doctors’ cognition on depressive symptoms in patients with epilepsy in Shanghai China.

**Methods:**

Questionnaires were handed out to doctors who have taken part in the epilepsy care, covering those from all third‐grade hospitals and several second‐grade hospitals in Shanghai China. Respondents were asked to make choices for their demographic profiles, clinical practices, acquired knowledge of, and attitudes toward the comorbidity of epilepsy and depression.

**Results:**

A total of 282 questionnaires were collected from 16 hospitals in Shanghai China, of which 280 copies were included in the statistical analysis. Respondents were mainly less than 50 years (260, 92.8%), mostly residents and attendings (206, 73.6%), and mostly master and doctor's degrees (225, 80.3%). The ratio of epileptologists and nonepileptologists was 56 (20.1%):224 (79.9%). Compared to nonepileptologists and residents, epileptologists and doctors with higher professional titles were more likely to answer that they received a higher percentage of patients with the comorbidity of epilepsy and depression (≥30%), and they knew very well about the knowledge, and held the view that depression exacerbated seizures (*p* < .05). Surprisingly, most doctors including chief doctors and epileptologists answered that they had difficulties in prescribing antidepressants. Quite a few doctors from lower class hospitals even preferred to use tricyclic antidepressants for controlling depressive symptoms in patients with epilepsy.

**Significance:**

Doctors, especially younger doctors and nonepileptologists, need more training to get knowledge of the comorbidity of epilepsy and depression. However, the therapeutic methods for depressive symptoms in patients with epilepsy were still limited and in a challenge.

## Graphical abstract

- A questionnaire‐based doctor survey was developed to investigate the knowledge of and attitudes towards the comorbidity of epilepsy and depression in Shanghai China.
- Epileptologists and doctors with higher professional titles have better knowledge and hold the view that depression exacerbates seizures.
- Most doctors including chief doctors and epileptologists answer that they have difficulties in prescribing antidepressants, indicating the therapeutic methods for depressive symptoms in patients with epilepsy are still limited and in challenge.

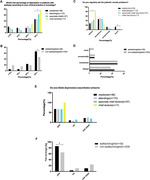

## 1 INTRODUCTION

Epilepsy is a chronic brain disorder characterized not only by recurrent seizures but also by its neurobiological, cognitive, psychological, and social consequences as pointed out by the International League Against Epilepsy (ILAE) in 2014 (Fisher et al., 2014). Psychiatric disorders are very common comorbidities in patients with epilepsy, of which depression has the highest prevalence (Tellez‐Zenteno et al., 2007). According to a recent meta‐analysis, the pooled prevalence of depressive disorders in patients with epilepsy until July 2016 was 22.9% (Scott et al., 2017). In some tertiary epileptic centers, the prevalence of depression in patients with epilepsy was even as high as 50% (Ring et al., 1998; Victoroff et al., 1994). A cross‐sectional study from four cities in China established that the prevalence rate of self‐reported depressive symptoms in patients with epilepsy was 24.1% (Fu et al., 2006).

Depressive symptoms in patients with epilepsy are usually relatively mild that might not meet the *Diagnostic and Statistical Manual* criteria of major depressive disorders (Krishnamoorthy et al., 2007), and symptoms such as suicidal idea, frustration intolerance, irritability, and motor agitation are unstable and alternated with symptom‐free periods. For this reason, Blumer et al. refer to it as interictal dysphoric disorder (IDD) (Blumer et al., 2004), and depressive symptoms in patients with epilepsy are usually unrecognized by clinicians due to the atypical features. For another, since most antidepressants are at a high risk of provoking seizures, especially when used in a rapid dose increase or at excessively high doses (Steinert & Froscher, 2018), the treatments for depressive symptoms in patients with epilepsy are still limited.

Increasing evidence shows that there are bidirectional relationships between epilepsy and depression (Hesdorffer et al., 2012; Josephson et al., 2017; Salpekar, 2017). A large‐scale clinical investigation by Hesdorffer et al. found that epilepsy was associated with an increased onset of psychiatric disorders and suicide before and after epilepsy diagnosis (Hesdorffer et al., 2012). An observational study of a population‐based cohort by Josephson et al. even found that treated depression (a surrogate for more severe depression than untreated depression) was associated with worse epilepsy outcome (Josephson et al., 2017). Clinically, seizure severity, seizure frequency, antiepileptic drugs (AEDs) taking, and social psychological factors were risk factors that promoted depression in patients with epilepsy (Peng et al., 2014; Thapar et al., 2005; Yildirim et al., 2018). Basic studies further demonstrated that common mechanisms including disturbance of neurotransmitters in the central nervous system, a hyperactive hypothalamic–pituitary–adrenal axis, brain structural changes, and inflammatory mechanisms were involved in the comorbidity of epilepsy and depression (Kanner et al., 2014).

Despite the high prevalence, depression in patients with epilepsy is commonly underdiagnosed and untreated (Kanner & Balabanov, 2002). The probable reasons in China are as follows: (1) Neurologists and neurosurgeons who take part in epilepsy care concern more about seizure control than underlying neuropsychiatric disorders. An online doctor survey on behalf of the Task Force of the ILAE Commission on Neuropsychiatry identified some key areas for improvement in managing the psychiatric comorbidities of epilepsy, suggesting that there are educational needs for clinicians taking part in epilepsy care in many countries (Mula et al., 2017). (2) The stigma of patients with epilepsy and their relatives may prevent them from turning to psychiatrists for help (Yildirim et al., 2018). Most patients are reluctant to see a psychiatrist or a psychologist in the specialized Mental Health Center in case of being looked in a peculiar way. To understand the current situation of attitudes toward and knowledge of the comorbidity of epilepsy and depression in doctors taking part in the epilepsy care in Shanghai, China, we designed a questionnaire in honor of the Shanghai Medical Association. Based on the questionnaire‐based doctor survey, we analyzed the underlying reasons for the undercognition of depression in patients with epilepsy.

## 2 METHODS

### 2.1 Questionnaire development

We conducted a literature review before designing the questionnaire and important key points were extracted from the literature. The keywords about the comorbidity of epilepsy and depression included as items in the questionnaire were as follows: the prevalence rate, risk factors, depression screening, and treatments. The questions in this questionnaire were classified into three categories: (1) the demographic profiles of respondents such as age, gender, educational degree, professional title, specialty, job role, and ranks of their hospitals; (2) the clinical practices of respondents for patients with epilepsy; (3) the knowledge about the prevalence, risk factors, relationships between epilepsy and depression, and therapeutic methods for depression in patients with epilepsy. The detailed items in the questionnaire are listed in the Supporting Information. The sponsor of this survey is Prof. Xin Wang who is the chairman of the epilepsy group affiliated to the Shanghai Medical Association.

### 2.2 Participants

There is a three‐level hierarchical medical system in Shanghai, which is representative of the way the health system is organized in big cities of China. The first‐grade hospitals are community hospitals that simply supply general practice services, therefore, most patients with epilepsy see doctors in the second‐grade and third‐grade hospitals that are mostly comprehensive hospitals and supply specialized medical services. Neurologists for adults, neurosurgeons, and psychiatrists who took part in the epilepsy care in all the tertiary hospitals, lower level third‐grade hospitals, and several main second‐grade hospitals in Shanghai, China, were covered in this survey. However, pediatric neurologists were not included in this survey because the psychiatric features of children with epilepsy might be different from adults. The participants completed the survey voluntarily.

### 2.3 Questionnaire handing out and recollecting

Doctors who were the standing committee members of the epilepsy group affiliated to the Shanghai Medical Association handed out the questionnaire in their hospitals. The same person recollected the questionnaires after 1 week.

### 2.4 Statistical data analysis

For the convenience of statistics, the multichoice options for these questions were set as categorical or ordinal variables, such as “yes/no/not clear,” “always/sometimes/occasionally/never,” and “<30%/30–60%/>60%.” The IBM SPSS version 21.0 (IBM Inc., Armonk, NY, USA) software was used to perform the statistical analysis. The chi‐square test or Fisher's exact test was conducted to compare parameters between specific groups, and the linear‐by‐linear association was adopted when the data fit the ordinal distribution. At last, a binary logistic regression was performed to establish the independent factors that affect doctors’ decision on whether to treat depressive symptoms in patients with epilepsy.

## 3 RESULTS

### 3.1 Demographic profiles of respondents

Doctors from 16 hospitals in Shanghai participated in the survey. In total, 282 questionnaires were returned, of which 280 copies were included in statistical analysis. Two questionnaires were excluded due to incompleteness. Demographic parameters of respondents are presented in Table 1. Most of the doctors were adult neurologists (253, 90.3%), and the others included neurosurgeons (9, 3.2%), psychiatrists (10, 3.6%), and traditional Chinese physicians (8, 2.9%). There were 225 of 280 doctors (80.4%) from tertiary hospitals, 21 of them (7.5%) from lower level third‐grade hospitals, and 23 of them (8.2%) from second‐grade hospitals in Shanghai. The ratio of male to female was 118(42.1%): 162(57.9%), almost equally distributed. Doctors were mainly less than 50 years (260, 92.8%), mostly residents and attendings (206, 73.6%), mostly master and doctor's degrees (225, 80.3%), and they were classified as epileptologists (56, 20.1%) and nonepileptologists (224, 79.9%) based on their subgroups of specialty.

**TABLE 1:** Demographic profiles of respondents

Total *n* = 280	*n* (%)
Gender	
Male	118(42.1%)
Female	162(57.9%)
Age (years)	
<30	77(27.5%)
30∼	102(36.4%)
40∼	81(28.9%)
50∼	20(7.2%)
Educational degree	
Bachelor	55(19.7%)
Master	149(53.2%)
Doctor	76(27.1%)
Professional title	
Residents	96(34.3%)
Attendings	110(39.3%)
Associate chief doctors	57(20.4%)
Chief doctors	17(6.1%)
Specialty	
Neurologists	253(90.3%)
Neurosurgeons	9(3.2%)
Psychiatrists	10(3.6%)
Doctors–traditional Chinese medicine	8(2.9%)
Epileptologists	56(20.1%)
Nonepileptologists	224 (79.9%)
Hospital	
Tertiary hospital	225(80.4%)
Other lower rank third‐grade hospital	21(7.5%)
Second‐grade hospital	23(8.2%)

### 3.2 Overall impression of doctors’ attitudes toward and cognition for the comorbidity of epilepsy and depression

All the doctors have taken part in epilepsy care, and 85.6% of them confirmed that they received epilepsy patients with depressive symptoms. About 37.4% of doctors said that they regularly asked the patients if they had any emotional disorders, most of the doctors (56.9%) sometimes or occasionally inquired the patients of this question, but there were still 5.7% of doctors never cared about the patients’ moods condition. When being asked whether the patients had complained of mood problems voluntarily, most of the doctors (75.7%) reported that less than 30% of patients with epilepsy did it. Over 50% of doctors believed that somatic symptoms, insomnia, and daytime somnolence were the three commonest complaints of depressive symptoms that had the highest prevalence of above 30%.

In the part of acquired knowledge, about 36.4% of doctors answered that they knew very little about the comorbidity of epilepsy and depression, and 2.9% of doctors reported that they had no idea of the related knowledge. Half of the doctors held the view that patients with generalized convulsive seizures would be more liable to have depression. Uncontrolled seizures (90%), inferiority or stigma (87.9%), and AEDs taking (40.4%) were the three major risk factors that were considered to exacerbate depressive symptoms in patients with epilepsy. Almost 70% of doctors thought depression and antidepressants might exacerbate seizures, and the depressive symptoms in patients with epilepsy should be controlled. However, 13.2% of doctors did not know which types of antidepressants they should prescribe, and 2.9% of doctors supposed the patients would reject the add‐on treatment with antidepressants. In the aspect of therapeutic methods, about 94.3% of doctors would recommend the patients to see a psychologist, over 40–50% of doctors would adjust the doses or types of AEDs, 73.5% of doctors would add on selective serotonin reuptake inhibitors (SSRIs), 30–40% of doctors would choose tricyclic antidepressants (TCAs) or traditional Chinese herbal antidepressants, and another 17.1% of doctors reported they would not take any measures to control the patients’ depressive symptoms. (The questionnaire is listed in the table as Supporting Information.)

### 3.3 Factors that affect doctors’ cognition and decision on treatments for the comorbidity of epilepsy and depression

**FIGURE 1:**
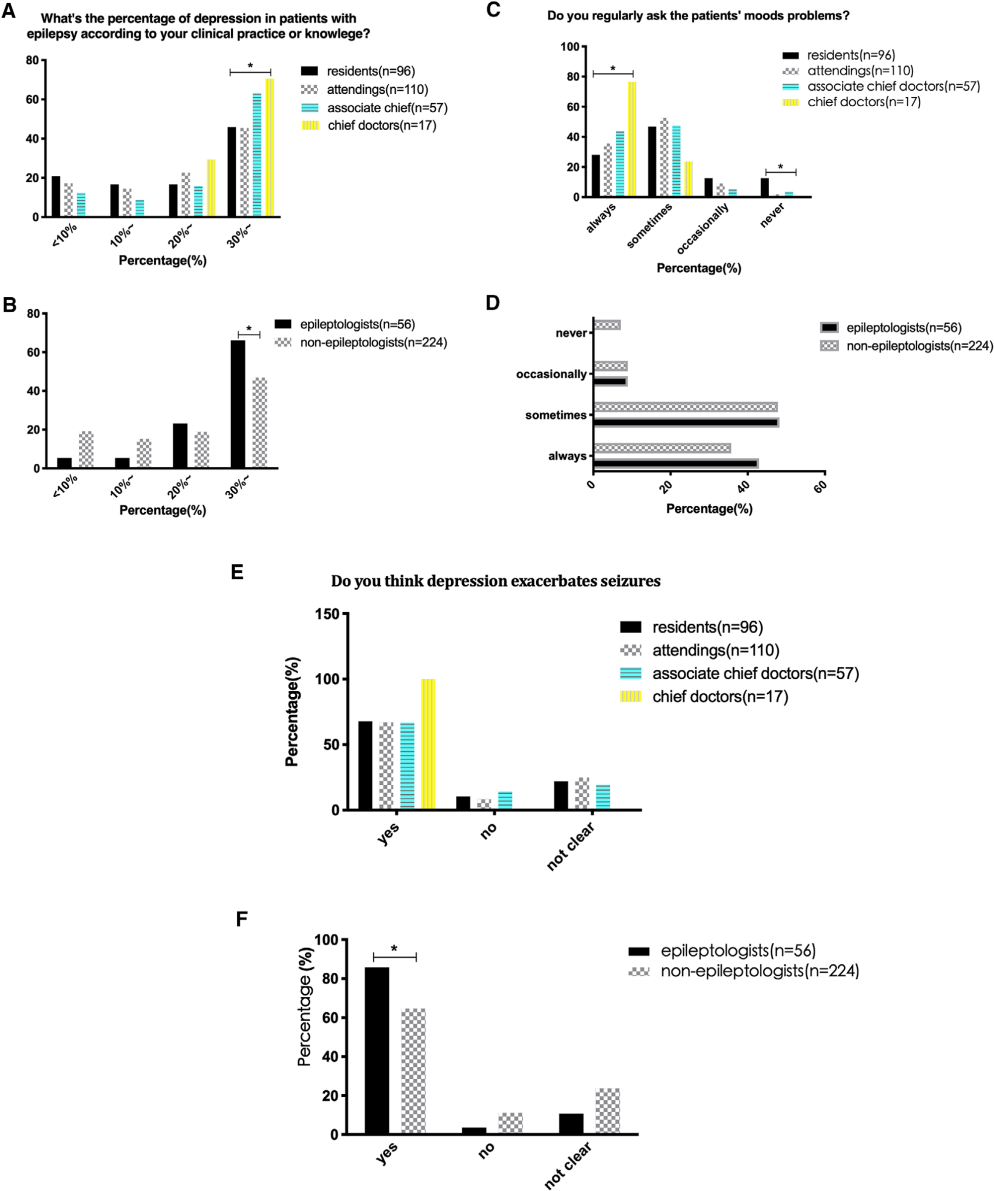
(a) Compared with attendings and residents, chief doctors and associate chief doctors were more likely to consider that the percentage of depression in patients with epilepsy was greater than 30% (*p < .05); (b) epileptologists chose that they received a higher percentage (30%∼) of patients with the comorbidity of epilepsy and depression than nonepileptologists (*p < .05); (c) most chief doctors chose that they always asked the patients’ moods problems, much higher than the residents (*p < .05); (d) no difference between epileptologists and nonepileptologists; (e) there were no differences among doctors with different professional titles who held the view that depression exacerbated epilepsy; (f) higher percentage of epileptologists believed depression exacerbated epilepsy compared with nonepileptologists (*p < .05)

**FIGURE 2:**
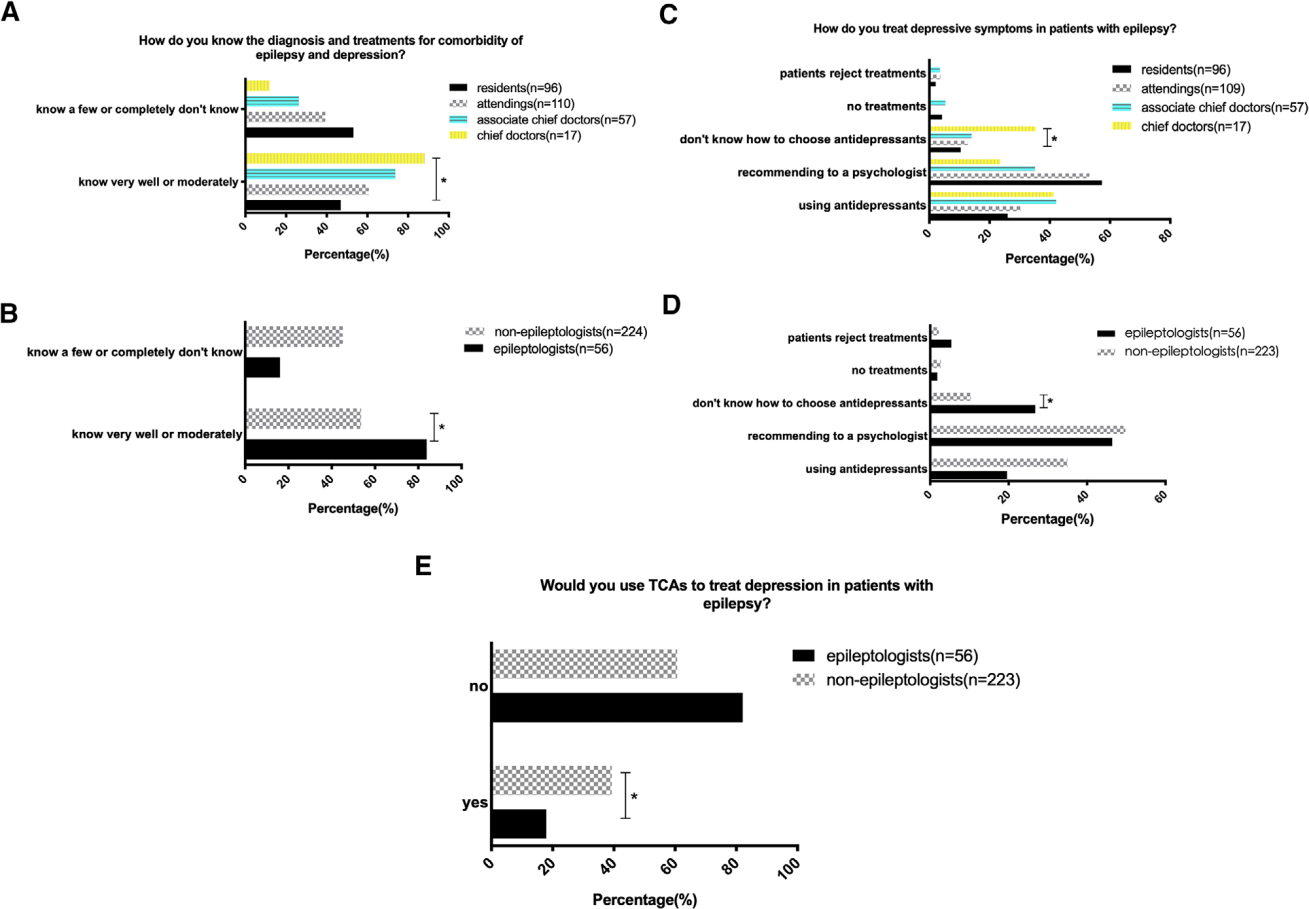
(a,b) As for the question “How well do you know the diagnosis and treatments for comorbidity of epilepsy and depression,” epileptologists and doctors with higher rank professional titles frequently chose “know very well or moderately,” while nonepileptologists, residents, and attendings mostly chose “know a few or completely do not know” (*p < .05); (c,d) most doctors chose to use antidepressants or recommend patients to a psychologist, but surprisingly, quite a few chief doctors including epileptologists chose that they did not know how to select antidepressants (*p < .05)

We compared the doctors’ opinions based on their specialties, professional titles, and the level of their hospitals. Compared with residents and attendings, chief doctors and associate chief doctors were more likely to think the percentage of depression in patients with epilepsy is greater than 30% (Figure 1a, *p* < .05). All chief doctors chose that they had received patients with the comorbidity of epilepsy and depression, while quite a few residents (27.4%) had not paid any attention to depressive symptoms in patients with epilepsy. Similarly, epileptologists had received a higher percentage of patients with the comorbidity of epilepsy and depression than nonepileptologists (Figure 1b, *p* < .05). For the question “Do you regularly ask the patients’ moods problems?” 76.5% of chief doctors chose that they always asked, much higher than the residents (28.1%); while 12.5% of residents answered they never asked, significantly higher than other subgroups of doctors (Figure 1c, *p* < .05). However, there was no difference for this question between epileptologists and nonepileptologists (Figure 1d), either in doctors from different level hospitals. Higher percentage of epileptologists held the view that depression exacerbated seizures compared with nonepileptologists (Figure 1f, *p* < .05), but there was no difference among subgroups of doctors with different professional titles (Figure 1e). As for “How well do you know the diagnosis and treatments for comorbidity of epilepsy and depression,” epileptologists and doctors with higher rank professional titles typically chose “know very well or moderately,” while nonepileptologists, residents, and attendings frequently answered “know very few or completely do not know” (Figure 2a,b; *p* < .05). For the therapeutic methods, most doctors prescribed antidepressants or recommended patients to a psychologist, but surprisingly, most doctors including epileptologists did not know how to choose antidepressants (Figure 2c,d; *p* < .05). Quite a large portion of nonepileptologists and doctors from lower class hospitals used TCAs for controlling depressive symptoms in patients with epilepsy compared to epileptologists and doctors from tertiary hospitals (*p* < .05).

At last, a binary logistic regression was conducted to establish the key factors that significantly affected doctors whether to prescribe antidepressants for controlling depression in patients with epilepsy. Whether doctors prescribed antidepressants for patients with comorbidity of epilepsy and depression was set as the dependent variable. The following independent factors were included for analysis that had significances performed by *t*‐tests: age (<30/30∼/40∼/50∼years), epileptologists or not (yes/no), percentage of patients with epilepsy they have received (<10%/10%∼/20%∼/30%∼), having received patients with the comorbidity of epilepsy and depression or not (yes/or), regularly asking patients’ moods problems (always/sometimes/occasionally/never), how well they know the diagnosis and treatments for the comorbidity of epilepsy and depression (very well or moderately/very few or completely do not know), whether antidepressants exacerbate seizures (yes/no), whether depression exacerbates seizures (yes/no/not clear), whether to use TCAs (yes/no). The step of “enter” was selected. The result showed that age, epileptologists or not, how well doctors know the diagnosis and treatments for the comorbidity of epilepsy and depression, and opinions on whether to use TCAs were key factors that mostly affected a doctor's decision on whether to use antidepressants for controlling depressive symptoms in patients with epilepsy (see Table 2).

**TABLE 2:** Key factors that affect a doctor's decision on whether to use antidepressants in patients with epilepsy

Variables in the equation^a^							
Age	0.786	0.306	6.608	1	0.01	2.195	1.205–3.998
Epileptologists or not	−1.143	0.464	6.079	1	0.014	0.319	0.129–0.791
How well the doctors know comorbidity of epilepsy and depression	1.329	0.369	12.957	1	0.000	3.779	1.832–7.793
Whether to use TCAs or not	0.869	0.305	8.131	1	0.004	2.384	1.312–4.331
Constant	−2.579	0.731	12.447	1	0.000	0.076	

^a^Conducted by the binary logistic regression.

## 4 DISCUSSION

This survey was performed in the name of the Shanghai Medical Association, China. The respondents were mostly from tertiary hospitals, occupying about 80%, and another 20% of doctors were also from medium‐scale comprehensive hospitals at different districts in Shanghai, so that the results of this survey could represent the viewpoints of most of the adult neurologists (including some doctors of Chinese herbal medicine), neurosurgeons who were engaged in epilepsy surgery, and psychiatrists in Shanghai. The results of this survey may have certain referential values for designing clinical studies and making related guidelines in China.

In the article written by Kanner et al., over 50% of depression in epilepsy has not been recognized by neurologists (Kanner & Balabanov, 2002), which might be attributed by multiple reasons. The first is that depressive symptoms in patients with epilepsy are sometimes not so typical and hardly differentiated with symptoms of seizures. It is reported that up to 50% of patients with the comorbidity of epilepsy and depression presented psychiatric symptoms that could not meet DSM or International Classification of Disease systems, which were referred to as IDD or peri‐ictal dysphoric syndrome (Mula, 2016). In this survey, the depressive symptoms of “insomnia,” “daytime somnolence,” and “somatic symptoms” were considered frequently occurred in patients with epilepsy, which had a consistency with the clinical study by Shen et al. (Shen et al., 2017), as insomnia was tightly associated with depression (Roberts & Duong, 2013), and treatments with AEDs might cause sleep disorders either (Jain & Glauser, 2014). The symptom of “impulsiveness” was considered to be popular in patients with epilepsy as well, indicating irritable temperament might be closely correlated with epilepsy (Erdogan Taycan & Taycan, 2014). Another reason for underdiagnosing depression in patients with epilepsy could be attributed to not enough attention paid by most doctors. In this survey, only 37.4% of doctors always asked whether the patients had mood problems, and even 5.7% of doctors never cared about the patients’ moods condition. It seemed that chief doctors paid more attention to patients’ mood problems than residents and attendings, while no difference was found among subgroups based on specialties and hospitals, indicating it is a common problem to neglect patients’ moods disorders by most younger doctors. Moreover, the percentage of patients with epilepsy complained of mood problems was relatively low (most doctors thought it is less than 30%), which might be due to stigma or inferiority.

Two main aspects of therapeutic methods for controlling depressive symptoms in patients with epilepsy have been recommended: pharmacological and psychological treatments. Except for antiseizure properties, AEDs have the effect of modulating moods and behavior as well (Perucca & Mula, 2013). Some of AEDs have been used as mood stabilizers such as valproate, lamotrigine, pregabalin, and clobazam, while some others are associated with mood deterioration or aggressive behavior (Brodie et al., 2016; Mula & Sander, 2007). Therefore, clinicians should thoroughly collect the psychiatric history and avoid prescribing AEDs that might aggravate mood problems. Adding on antidepressants is another choice of pharmacological therapy. SSRIs and serotonin and norepinephrine reuptake inhibitors are first recommended in patients with epilepsy (Mula, 2017), but still with potential risks of increasing seizures. Sertraline and citalopram are considered to be the first‐line choice, since other SSRIs such as fluoxetine may have pharmacological interactions with AEDs (Mula et al., 2008). Traditional antidepressants such as TCAs may induce epileptic discharges in electroencephalographic studies, especially when taken in high dose (>200 mg) (Alper et al., 2007; Mula, 2017), which are not recommended to be used in patients with epilepsy. Cognitive‐behavioral therapy and mind–body interventions are common psychological treatments for patients with epilepsy and demonstrated to be effective in improving psychological well‐being and seizure control (Tang et al., 2014). A meta‐analysis supplied a moderate‐quality evidence that psychological treatments enhanced health‐related quality of life for adults with epilepsy (Michaelis et al., 2018). In this survey, most doctors recommended patients to see psychologists or add on SSRIs, while there were still 35% of doctors who use TCAs, especially in nonepileptologists and doctors from lower level hospitals. The Chinese herbal antidepressant *Xylaria* nigripes has been demonstrated to have moderate antidepressant effects and do not exacerbate seizures in patients with epilepsy (Peng et al., 2015). About 41.8% of doctors used traditional Chinese herbal antidepressants. However, surprisingly, although chief doctors and epileptologists generally considered depression exacerbated seizures and had good knowledge of comorbidity of epilepsy and depression, quite a few of them answered they did not know how to choose antidepressants. We believe that this phenomenon reflects the dilemma of the current situation that most doctors have difficulties in prescribing antidepressants for patients with epilepsy due to the risk of exacerbating seizures. Furthermore, there are still limited therapeutic methods for controlling depressive symptoms in patients with epilepsy, which raises the challenge for most doctors in the future.

At last, in our study, factors that affected doctors’ decision on whether to use antidepressants to treat depression in patients with epilepsy were as follows: age, epileptologists or not, how well doctors know the diagnosis and treatments for the comorbidity of epilepsy and depression, and opinions on whether to use TCAs. As age is positively correlated with doctors’ professional titles, it is reasonable that doctors with elder age have more experiences using antidepressants than younger doctors. It is worth mentioning that nonepileptologists prefer to prescribe antidepressants than epileptologists, but they use TCAs frequently, which indicates that they do not have as much knowledge of the comorbidity of epilepsy and depression as epileptologists.

## 5 CONCLUSIONS

This questionnaire‐based investigation indicates that most doctors taking part in epilepsy care, especially epileptologists, realize that there is a high prevalence of the comorbidity of epilepsy and depression, depression exacerbates seizures, and depressive symptoms should be controlled in patients with epilepsy. Epileptologists have more knowledge about the comorbidity of epilepsy and depression than nonepileptiologists. However, how to choose antidepressants is still a dilemma for most doctors including epileptologists. This survey in Shanghai reflects the current condition of diagnosis and treatments for the comorbidity of epilepsy and depression in big cities of China, which highly indicates that making related guidelines may contribute to set a consensus for doctors to recognize and treat depressive symptoms in patients with epilepsy.

## CONFLICT OF INTEREST

The authors declare that there is no conflict of interest.

6

### PEER REVIEW

The peer review history for this article is available at https://publons.com/publon/10.1002/brb3.2232.

## Supporting information

Supporting Information

Supporting Information

## Data Availability

The data that support the findings of this study are available from the corresponding author upon reasonable request.
