# Supplementary material for: A survey on doctors’ cognition of depression in patients with epilepsy
Source: Brain Behav. 2021 Jun 4;11(8):e2232. doi: 10.1002/brb3.2232 (PMC8413820; doi:10.1002/brb3.2232)
Supplement: Supplementary file 1 — Supporting Information [file BRB3-11-e2232-s001.docx]

**Appendix1: Authors and contributions**

| **Name** | **Location** | **Contribution** |
| --- | --- | --- |
| Xin Wang | Zhongshan Hospital, Fudan University | Design and conceptualized the investigation |
| Jing Ding | Zhongshan Hospital, Fudan University | Design and organized the investigation |
| Weifeng Peng | Zhongshan Hospital, Fudan University | Major role in the acquisition of data and drafted the manuscript for intellectual content |
| Shaokang Zhan | Shanghai Medical College, Fudan University | Analyzed the data |

**Appendix 2: Co-investigators**

| **Name** | **Location** | **Contribution** |
| --- | --- | --- |
| Guoxing Zhu; Xunyi Wu | Huashan Hospital, Fudan University | Handed out questionnaires |
| Qinchi Lu; Hongyu Zhou | Renji Hospital affiliated to Shanghai Jiaotong University Medical School | Handed out and re-collected questionnaires |
| Zhi Gen | Shanghai Sixth People’s Hospital affiliated to Shanghai Jiaotong University Medical School | Handed out and re-collected questionnaires |
| Min Zhu | Shanghai First People’s Hospital affiliated to Shanghai Jiaotong University Medical School | Handed out and re-collected questionnaires |
| Lin Li | Xinhua Hospital affiliated to Shanghai Jiaotong University Medical School | Handed out and re-collected questionnaires |
| Zhengqin Zhao | Changzheng Hospital affiliated to Naval Medical Universtiy | Handed out and re-collected questionnaires |
| Yulei Deng | Ruijin Hospital affiliated to Shanghai Jiaotong University Medical School | Handed out and re-collected questionnaires |
| Yujuan Chen | Shanghai Tenth People’s Hospital affiliated to Tongji University | Handed out and re-collected questionnaires |
| Yuhui Chen; Qingwei Li | Tongji Hospital affiliated to Tongji University | Handed out and re-collected questionnaires |
| Gang Li | Dongfang Hospital affiliated to Tongji University | Handed out and re-collected questionnaires |
| Zheng Jin | Shanghai Fifth People’s Hospital affiliated to Fudan University | Handed out and re-collected questionnaires |
| Zhigang Dong | Shanghai Eighth People’s Hospital | Handed out and re-collected questionnaires |
| Jie Chen | Yueyang Hospital of Integrated Traditional Chinese and Western Medicine, Shanghai University of Traditional Chinese Medicine | Handed out and re-collected questionnaires |
| Weixia Yang | Qingpu District Central Hospital | Handed out and re-collected questionnaires |
