# Supplementary material for: A survey on doctors’ cognition of depression in patients with epilepsy
Source: Brain Behav. 2021 Jun 4;11(8):e2232. doi: 10.1002/brb3.2232 (PMC8413820; doi:10.1002/brb3.2232)
Supplement: Supplementary file 2 — Supporting Information [file BRB3-11-e2232-s002.docx]

**Table** Items of the questionnaire and general opinions of doctors on the comorbidity of epilepsy and depression

| Total n=280 | n (%) |
| --- | --- |
| **Doctors’ attitudes towards comorbidity of epilepsy and depression** | |
| **Have you received patients with the comorbidity of epilepsy and depression?**  Yes, I received epilepsy patients with definite depression  Yes, I received epilepsy patients with suspicious depression  No, I never saw patients with comorbidity of epilepsy and depression  I haven’t pay attention to epilepsy patients’ moods | 141(50.4%)  101(36.1%)  20(7.1%)  16(5.7%) |
| **Have you regularly asked the patients moods problems?**  Always  Sometimes  Occasionally  Never | 104(37.4%)  134(47.9%)  25(9.0%)  16(5.7%) |
| **What’s the percentage of patients with epilepsy complained of moods problems in your clinical practice?**  <30%  30~60%  >60% | 212(75.7%)  56(20%)  10(3.6%) |
| **What’s the percentage of the following complains for depressive symptoms in the patients with epilepsy you have seen?**   1. Depressed moods   <30%  30~60%  >60%  2. Suicide ideas  <30%  30~60%  >60%  3. Somatic symptoms  <30%  30~60%  >60%  4. Insomnia  <30%  30~60%  >60%  5. Daytime somnolence  <30%  30~60%  >60%  6. Impulsiveness  <30%  30~60%  >60%  7. Restlessness  <30%  30~60%  >60% | 170(60.7%)  85(30.4%)  24(8.6%)  267(95.4%)  10(3.6%)  2(0.7%)  121(43.2%)  121(43.2%)  37(13.2%)  129(46.1%)  126(45%)  23(8.2%)  111(39.6%)  129(46.1%)  39(13.9%)  149(53.2%)  113(40.4%)  16(5.7%)  168(60%)  100(35.7%)  9(3.2%) |
| **Doctors’ cognition for the comorbidity of epilepsy and depression** | |
| **How do you know the diagnosis and treatments for the comorbidity of epilepsy and depression?**  Know it very well or have taken part in related courses or trainings  Know a little or have read some literatures  Don’t know much  Completely don’t know | **65(23.2%)**  102(36.4%)  102(36.4%)  8(2.9%) |
| **Do you think which type of seizure is more liable to be comorbid with depression?**  Generalized convulsive seizure  Focal seizure with impaired awareness  Focal seizure with unimpaired awareness  Other seizure types  Not clear | 141(50.3%)  70(24.9%)  37(13.2%)  2(0.7%)  40(14.4%) |
| **What are the commonest factors promoting depression in patients with epilepsy? Please list the most important three factors.**  Uncontrolled seizures  Inferiority and stigma  AEDs  Difficulties in finding jobs  Unhappy family life  Recent psychiatric stress  Difficulties in economy | 252(90%)  246(87.9%)  113(40.4%)  106(37.9%)  79(28.2%)  34(12.1%)  31(11.1%) |
| **What kinds of treatments would you use when you see patients with comorbidity of epilepsy and depression?**   1. Change the types of AEDs   Yes  No   1. Change the doses of AEDs   Yes  No   1. Add SSRIs antidepressants   Yes  No   1. Add tricyclic antidepressants   Yes  No   1. Add traditional Chinese herbal antidepressants   Yes  No   1. Add benzodiazepine   Yes  No   1. Recommend patients to see a psychiatrist or psychologist   Yes  No   1. No treatments   Yes  No | 124(44.3%)  156(55.7%)  142(50.7%)  138(49.3%)  207(73.9%)  71(25.4%)  **98(35%)**  182(65%)  117(41.8%)  163(58.2%)  171(61.1%)  109(38.9%)  **264(94.3%)**  16(5.7%)  **48(17.1%)**  232(82.9%) |
| **Do you think depression exacerbate seizures?**  Yes  No  Not clear | 193(68.9%)  27(9.6%)  59(21.1%) |
| **Do you think antidepressants exacerbate seizures?**  Yes  No  Not clear | 221(78.9%)  48(17.1%)  7(2.5%) |
| **Do you think the depressive symptoms need to be treated in patients with comorbidity of epilepsy and depression?**  Yes, it need to be treated with antidepressants  Yes. But the patient should be transferred to psychology department  No, it’s not severe and no need to be treated  It needs to be treated but I don’t know what kinds of medicines can be used  The patients always reject to be treated | 85(30.4%)  **137(48.9%)**  7(2.5%)  **37(13.2%)**  8(2.9%) |
